# Isolation and Characterization of Porcine Amniotic Fluid-Derived Multipotent Stem Cells

**DOI:** 10.1371/journal.pone.0019964

**Published:** 2011-05-19

**Authors:** Jiahuan Chen, Zhijuan Lu, De Cheng, Sha Peng, Huayan Wang

**Affiliations:** 1 Department of Animal Biotechnology, College of Veterinary Medicine, Northwest A&F University, Yangling, Shaanxi, People's Republic of China; 2 Zhengzhou Senior High School, Zhemgzhou, Henan, People's Republic of China; Pennington Biomedical Research Center, United States of America

## Abstract

The aim of this study was to isolate and characterize porcine amniotic fluid-derived multipotent stem cells (pAF-MSC). The porcine amniotic fluid (AF) from the amniotic cavity of pregnant gilts in the early stages of gestation (at E35) was collected and centrifuged for 5–10 min at 400 g to pellet cells. The primary culture of AF showed the multiple cell types, including the epithelial-like cells and fibroblast-like cells. By culturing in AMM medium for 6 to 8 days, the epithelial-like cells disappeared and the remaining cells presented the fibroblastoid morphology. The doubling time of pAF-MSCs was about 34.6 h, and the cells had been continually cultured over 60 passages in vitro. The flow cytometry results showed that pAF-MSCs were positive for CD44, CD117 and CD166, but negative for CD34, CD45 and CD54. Meanwhile, pAF-MSCs expressed ES cell markers, such as Oct4, Nanog, SSEA4, Tra-1-60 and Tra-1-81. The ratio of CD117^+^ CD44^+^ cells accounted for 98% of pAF-MSCs population. Three germ layer markers, including FGF5 (ectodermal marker), AFP (endodermal marker) and Bra (mesodermal marker), were detected in embryoid bodies derived from pAF-MSCs. Under the different induction conditions, the pAF-MSCs were capable of differentiating into neurocytes, adipocytes and beating cardiomyocytes. Furthermore, the pAF-MSCs didn't form teratoma when injected into immunodeficiency mice. These optimal features of pAF-MSCs provide an excellent alternative stem cell resource for potential cell therapy in regenerative medicine and transgenic animals.

## Introduction

Amniotic fluid (AF) is composed of normal embryonic or fetal chipping cells derived from the three germ layers (ectoderm, endoderm and mesoderm) [1], [2]. Therefore, it possesses the natural precursors of all differentiation lineages. Recently, AF has been used as a source of human mesenchymal stem cells (hMSCs) that express the specific markers, such as CD90, CD105, CD73 and CD166 [3], [4], [5], [6]. Meanwhile, Oct4-expressing cells are present in human amniotic fluid [7], indicating that human amniotic fluid may be a new source of pluripotent stem cells without any ethical concerns associated with human embryonic stem cells (hES cells) research [8], [9].

The cells isolated from human amniotic fluid were first named human amniotic fluid stem cells (hAFSCs) in 2007 [4]. Over 90% of hAFSCs express Oct4 (the specific marker of ES cells). Besides, CD117 is also used as a critical marker for identifying AFS cells [1]. The hAFSCs have self-renewal capability and multi-lineage differentiation potential similar to ES cells. It has been reported that human AFS cells could differentiate into multiple cell lineages, including adipocytes [5], osteoblasts [4], chondrocytes [10], cardiomyocytes [1], [11], [12], [13], endothelial cells [4], neurocytes [14], hepatocytes [4] and renal cells [11]. However, unlike ES cells, the hAFSCs don't form teratoma when injected subcutaneously into nude mice [7]. Thus, the AFS may be an intermediate type of cells between ES cells and adult stem cells.

Pig is a main domestic animal and has many biophysical and biochemical similarity to human beings. The use of pig models for pre-clinical testing is well established and the availability of embryonic stem cells may open the way to pre-clinical experimentation for any kind of cell therapy. However, thus far, the stable embryonic stem cell lines have not yet been generated from pig embryos, but rather obtained by normal fertilization or by parthenogenetic activation [15]. The iPS technology has been used to generate pig iPS cells that presented the defining features similar to either human or mouse ES cells [16], [17]. Although the chimera pig derived from pig iPS cells has been reported [18], several important issues remain to be addressed, such as the low efficacy of generating fully reprogrammed pig iPS cell line and the safety of iPS cells. Therefore, the alternative stem cells such as porcine amniotic fluid mesenchymal stem cells (pAF-MSCs) may provide a novel and convenient cell resource, which can be used for transgenic animal research and regeneration medicine.

In this paper, we characterized pAF-MSCs and demonstrated the multipotency of pAF-MSCs. Our study revealed that these cells were similar to hAFSCs and could differentiate into cell derivatives of the three germ layers, especially the beating cardiomyocytes.

## Results

### The pAF-MSCs isolated from porcine amniotic fluid

**Figure 1:**
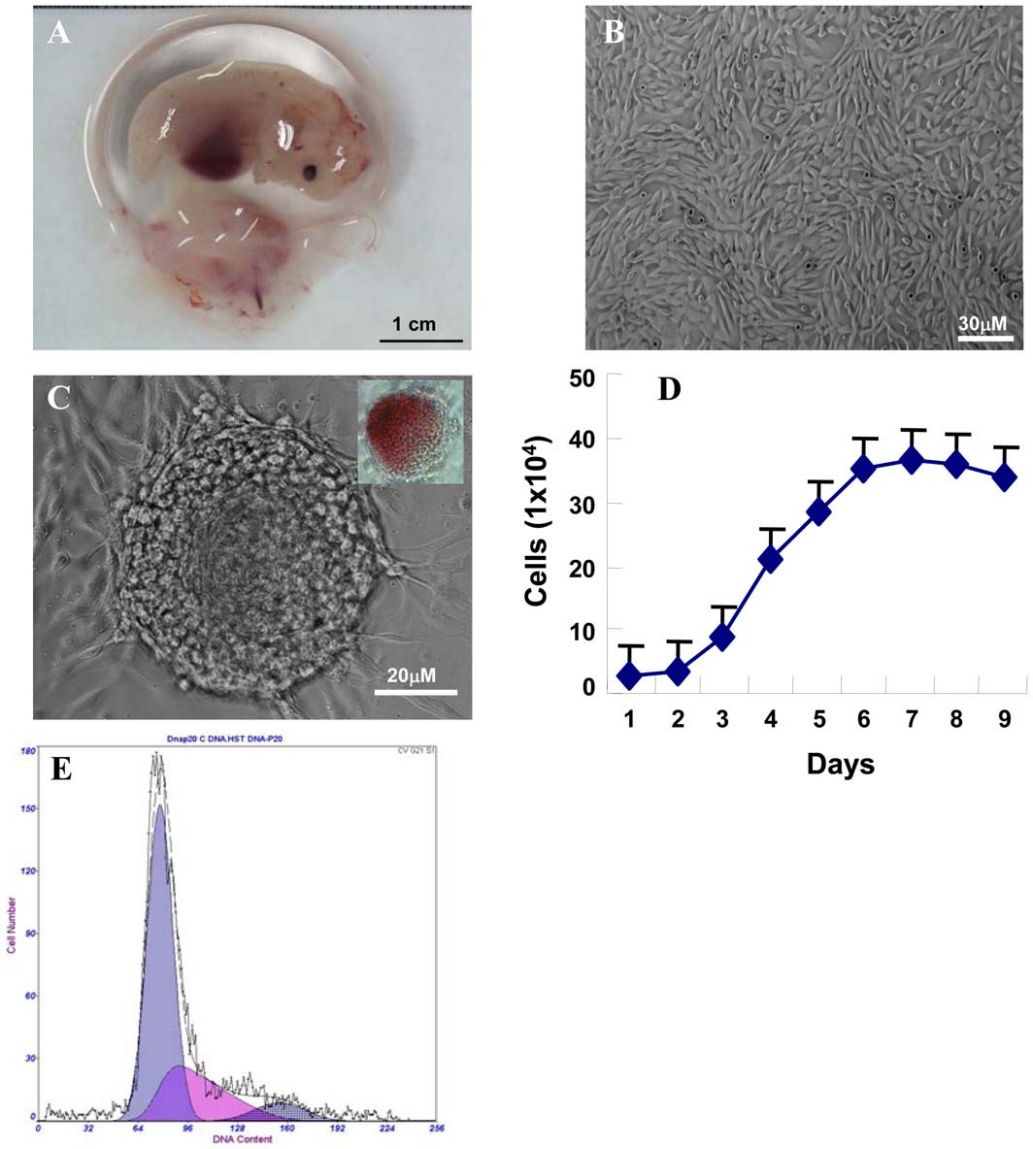
Isolation of pAF-MSCs from the amniotic fluid of E35 porcine fetus. (A) The E35 porcine fetus with whole amniotic membrane. The amniotic fluid was collected under sterile condition. (B) The morphology of pAF-MSCs after culturing for 3 passages *in vitro*. Most cells exhibited fibroblast-like shape. (C) ES cell-likes colony appeared occasionally in the primary pAF-MSCs culture, which was AP staining positive (inset). (D) The growth curve of the pAF-MSCs (at 6^th^ passage). The doubling time was 34.6 h. (E) The cell cycle of pAF-MSCs was analyzed by FACS.

The porcine AF was collected from amniotic cavity in the early stage gestation of porcine fetus (at E35, Fig. 1A). After 3 days of in vitro culture, both fibroblast-like cells and epithelioid cells appeared. For 2 to 3 passages, most cells exhibited fibroblast-like phonotype (Fig. 1B), and occasionally, some ES cell-like colonies were observed and these colonies were positive for AP staining (Fig. 1C). In early culture passages (0 to 3) due to the existing mixed cell populations, the cell growth rate was slow, which took 3–4 days to passage once. After 4–5 passages, the cell populations exhibited the uniform fibroblast-like morphology, and the growth rate of pAF-MSCs had the doubling time of 34.6 h (Fig. 1D), which was quite similar to the doubling time of human hAFSCs. The result of the cell cycle analysis showed that 27.3% of cells were in the S phase (Fig. 1E). For routine culture, cells were grown in the 3.5 cm culture plate, and when reached 80% confluence (0.8×10^6^ cells/plate), cells in one plate were split into three 3.5 cm plates (0.25×10^6^ cells/plate).

Six pAF-MSC cell lines were frozen at passages 8–10. Two cell lines, which showed the similar cell surface markers of CD117+ and CD44+, were cultured for more than 20 passages. One cell line was frozen at passage 23 and one cell line, which was used in this study, was continuously cultured for 60 passages.

### Characterization of pAF-MSCs

**Figure 2:**
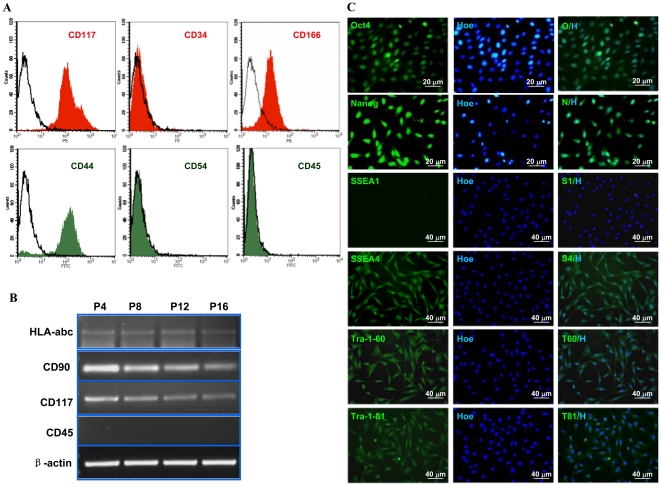
Determination of specific gene markers in pAF-MSCs. (A) The expression of cell surface antigens was examined by flow cytometry. The antibodies of CD34, CD117 and CD166 were labeled with PE, and CD45, CD44 and CD54 were labeled with FITC. (B) The mRNA expressions of CD90, CD117 and HLA-abc, but not CD45 were detectable in pAF-MSCs by semi-quantitative RT-PCR assay. The RNA samples were prepared from pAF-MSCs at 4^th^, 8^th^, 12^th^ and 16^th^ passages. The β-actin was used as internal control. (C) The pluripotent markers of ES cell were determined in pAF-MSCs (at 6^th^ passage) by immunofluorescence assay. The antibodies against Oct4, Nanog, SSEA1, SSEA4, Tra-1-60 and Tra-1-81 were conducted and the positive staining showed green fluorescence (FITC). The nuclei were stained by Hoechst 33342 (blue fluorescence).

It has been reported that human AFS cells expressed the surface antigens including CD117, CD44, CD90, and CD29, but not CD45 and CD34 [13]. Based on the flow cytometry and RT-PCR analyses, we found that the pAF-MSCs expressed CD117, CD44, CD166 (Fig. 2A) and HLA-abc, CD90 (Fig. 2B), but not CD34, CD45, and CD54 (Fig. 2A, B). The ratio of CD117^+^/CD44^+^ cells accounted for 98% of pAF-MSCs population. The immunofluorescence analysis of ES specific markers showed that pAF-MSCs expressed Oct4, Nanog, SSEA4, Tra-1-60 and Tra-1-81, but no expression of SSEA1, demonstrating that pAF-MSCs had the similar gene expression profiles as described in human ES cells (Fig. 2C). These results revealed that the isolated pAF-MSCs might be at the pluripotent cell transition from the embryonic stem cell phase to the mesenchymal stem cell phase since pAF-MSCs expressed both ES and MSC specific markers.

### The multi-lineage differentiation of pAF-MSCs

**Figure 3:**
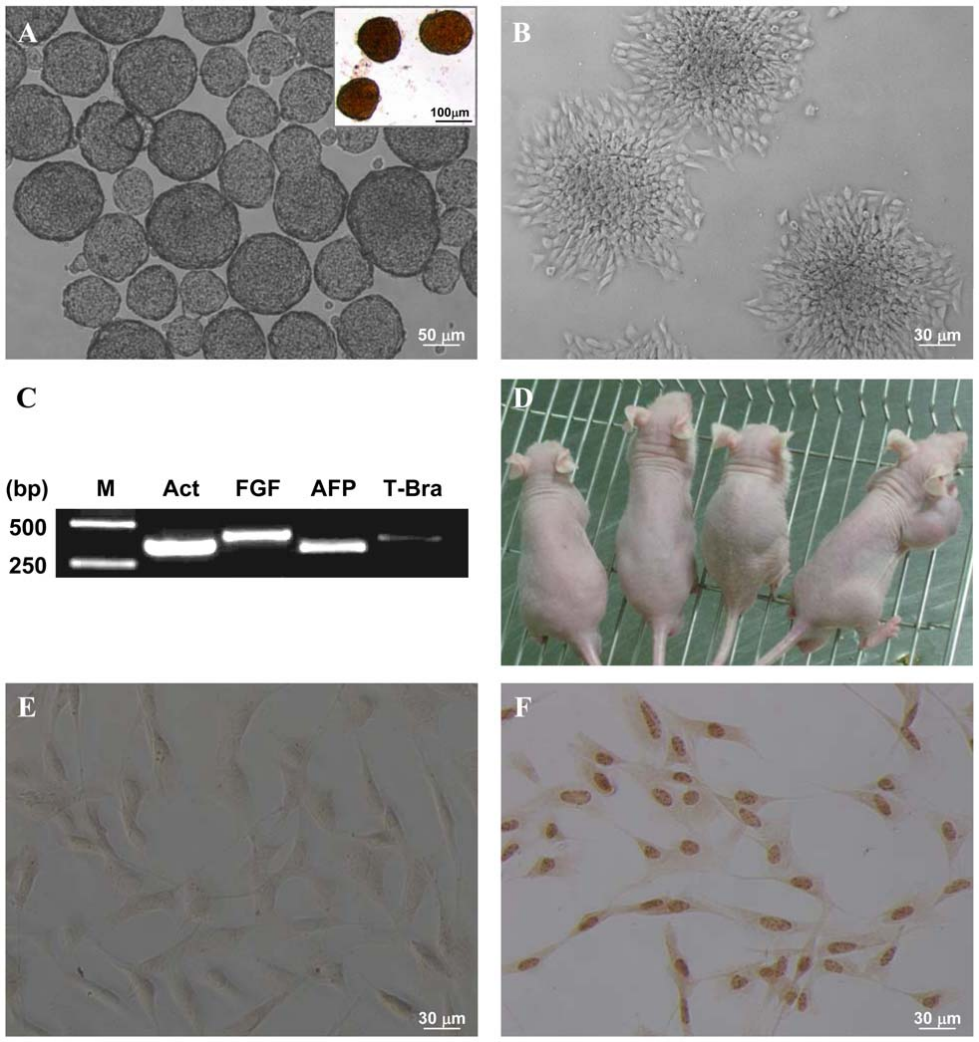
The EBs and teratoma formation. (A) The pAF-MSCs were cultured in suspension at the uncoated Petri dishes for EBs formation. After 24 h of culture EBs were formed and showed positive for alkaline phosphatase (AP) staining (inset). (B) When EBs cultured in the adherent dish for 24 h, the differentiated cells were observed. (C) The expression of three germ layers markers were detected by RT-PCR in the differentiated cells from EBs. M, DNA marker; Act, β-actin used as an internal control; FGF, Fibroblast growth factor 5 for the primitive ectoderm; AFP, Alpha fetoprotein for the endoderm; T-Bra, T-box gene brachyury for the mesoderm. (D) The pAF-MSCs (5×10^6^ cells/injection) were subcutaneously injected into (BALB/c-Nu) the right axilla of three immunodeficient mice. One mouse injected with mES J1 cells (2×10^6^ cells/injection) was as positive control. After six weeks post-injection, three mice injected with pAF-MSCs (on the left) were no teratomas, but mouse injected with mES J1 cells (on the right) showed the teratoma. (E–F) The TERT expression was detected by anti-TERT antibody in pig embryonic fibroblasts (E) and pAF-MSCs (F).

The reports showed that during the formation of embryoid bodies (EBs) without the treatment with inducers, stem cells were able to spontaneously differentiate [19]. To determine the differentiation potential, the pAF-MSCs were grown in suspension to form EBs (Fig. 3A–B). We then examined the expression of three embryonic germ layers' marker in EBs by RT-PCR assay. The transcriptional expression of FGF5 (a specific marker of ectoderm), AFP (a specific marker of endoderm) and Bra (a specific marker of mesoderm) were detectable, indicating that pAF-MSCs were capable of differentiation in vitro (Fig. 3C). To check the tumorigenicity of pAF-MSCs, the cells were subcutaneously injected into three nude mice (BALB/C-nu). One mouse injected with mES J1 cells was used as the positive control. As shown in Figure 3D, no teratoma was observed in three mice injected with pAF-MSCs, but the teratoma appeared in the mouse injected with mES J1 cells (Fig. 3D). This observation suggested that the pAF-MSCs, unlike ES cells, didn't form teratoma and might be much safer to be used in cell therapeutic application. We also determined the telomerase reverse transcriptase (TERT) expression, which is one of essential telomerase components and plays the key role to maintain the telomere length of the chromosome in pAF-MSCs. The results showed that TERT solely presented in pAF-MSC cell nucleus, but was absent in pig embryonic fibroblasts (PEF, Fig. 3E–F).

**Figure 4:**
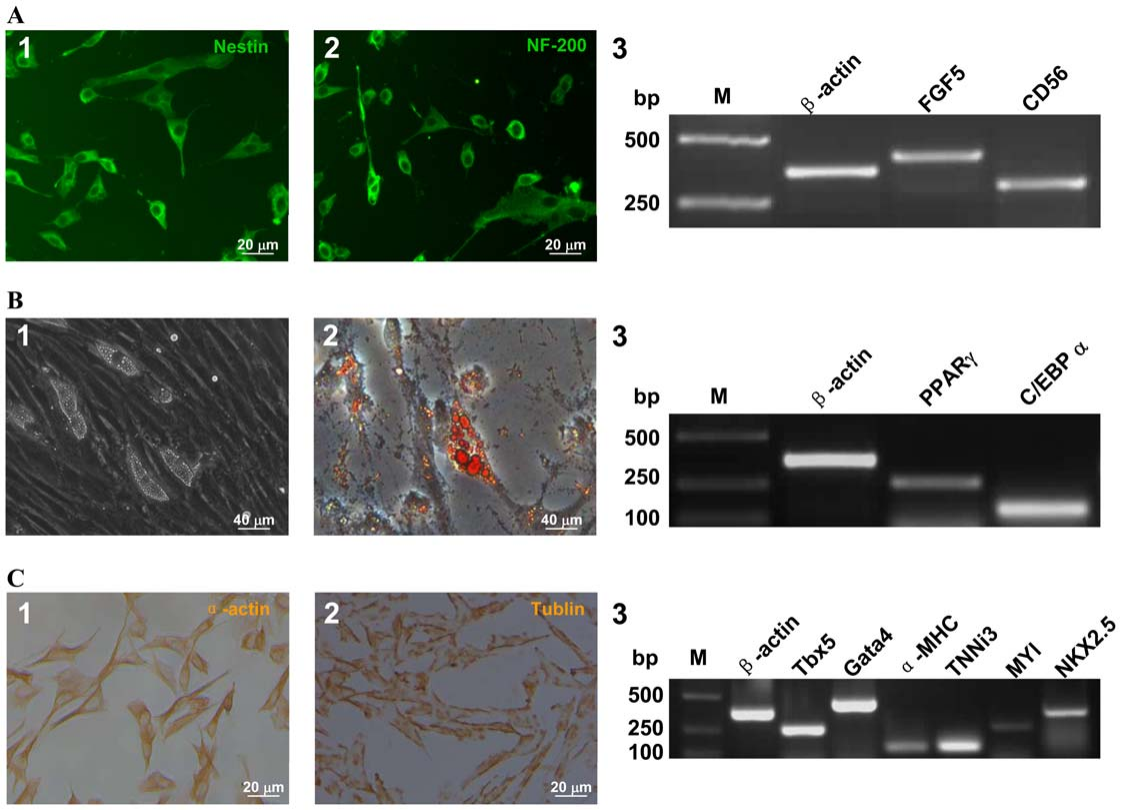
The multi-lineage differentiation potential of pAF-MSCs *in vitro*. The specific markers were detected by immunocytochemistry (ICC) and RT-PCR. (A) The pAF-MSCs differentiated into neurocytes. (1) ICC of Nestin; (2) ICC of NF-200; (3) RT-PCR assay for FGF5 and CD56. (B) The pAF-MSCs differentiated into adipocytes. (1) The lipocytes were induced for two weeks. The lipid droplets were round the nuclei (inset); (2) The cells were positive for Oil Red O staining; (3) RT-PCR assay for PPARγ and C/EBPα in adipocyte derived from pAF-MSCs. (C) The pAF-MSCs differentiated into cardiomyocytes. (1) ICC of α-actin; (2) ICC of ß-tublin; (3) RT-PCR assay for cardiomyocyte specific markers including Tbx5, Gata4, α-MHC, TNNi3, MYL and Nkx2.5. Act, β-actin was used as the internal control; M, DNA marker.

Furthermore, to evaluate the multi-lineage differentiation potential, the pAF-MSCs at the 12^th^ passage were cultured in different induction conditions. After incubation with the neurogenic differentiation medium, cells differentiated toward neuron-like cells and expressed neuron specific markers, including Nestin, NF-200, FGF5 and CD56 (Fig. 4A). With the treatment of dexamethasone, methylxanthine, insulin and indomethacin, cells were differentiated into adipocytes that looked like white fat cells containing a large number of lipid droplet surrounded by a layer of cytoplasm (Fig. 4, B1). The adipocytes were positively stained by Oil Red O, and expressed adipose specific markers of PPARγ and C/EBPα (Fig. 4, B2-3). When pAF-MSCs were cultured in the inducing medium that contained Vitamin C and 5-aza, cells were differentiated towards cardiomyocytes. The cardiac-specific markers were detected in the differentiated pAF-MSCs, including α-actin, β-tublin, TNNi3, TbX5, Gata4, α-MHC, MYL and Nkx2.5 (Fig. 4C). These observations indicated that the pAF-MSCs possessed the multipotency and had the potential to differentiate into three germ layers *in vitro*.

### The pAF-MSCs differentiate into the beating cardiomyocytes

**Figure 5:**
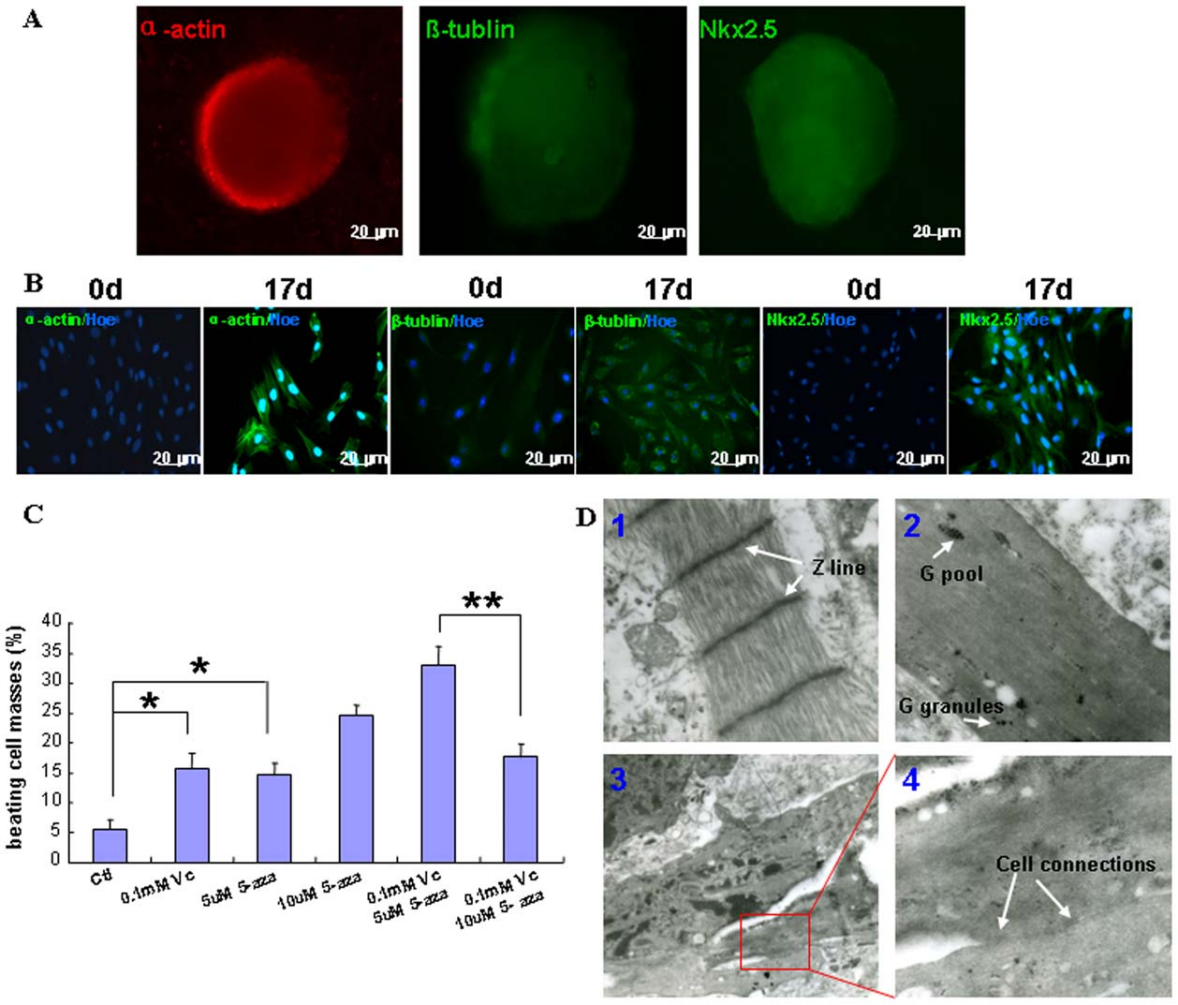
The pAF-MSCs differentiated into beating cardiomyocytes in vitro. (A) The EBs cultured in suspension with cardiac differentiation medium for 17 day expressed cardiomyocyte special markers, including α-actin, β-tublin and Nkx2.5. (B) The pAF-MSCs continually grew in cardiomyogenic medium for 17 days in the tissue culture plate to induce the differentiation into cardiomyocytes. The immunofluorescence analysis was conducted to determine α-actin, ß-tublin and Nkx2.5 expression in the uninduced pAF-MSCs (0 d) and the induced pAF-MSCs (17 d). (C) The percentage of beating cell masses induced by the different media was counted. * indicates difference at p<0.05; ** indicates significant difference at p<0.01. (D) Transmission electron microscope analysis of the beating cell masses. 1, pig heart tissue was used as the control. The arrows indicate the Z lines (×25 k); 2, the cardiac-muscle like filament from the beating cell masses derived from pAF-MSCs. The arrows indicated the glycogen pool and glycogen granules (×30 k). 3, the cardiac-muscle like structure was observed in the beating cell masses (×12 k). 4, The enlarged photo showed the cell connections (arrows) (×50 k).

The EBs derived from pAF-MSCs expressed cardiac-special markers, including α-actin, β-tublin, and Nkx2.5 (Fig. 5A). In two-three weeks induction, the cell masses derived from pAF-MSCs started to beat rhythmically (Fig. S1, Movie S1, S2). We also tried the method for directing the commitment of pAF-MSCs from monolayer to cardiac muscle. Although the beating cells were observed in some cases, the efficiency of cardiac induction was low (Fig. S1, Movie S3). We then kept away from this inducing method. The results of immnuocytochemistry assay showed that the differentiated cells strongly expressed cardiac specific markers (α-actin, β-tublin and Nkx2.5), while the undifferentiated pAF-MSCs only weakly expressed ß-tublin, but not α-actin and Nkx2.5 (Fig. 5B).

To determine the induction efficiency, five differentiation media were used to induce pAF-MSCs differentiation towards cardiomyocytes. In the control, the spontaneously differentiated beating cells were in 5%. With the treatment of either 0.1 mmol/L Vc or 5 µmol/L 5-aza, the beating cell numbers were significantly increased (Fig. 5C). When the concentration of 5-aza was elevated to 10 µM, the beating cell numbers were further increased, suggesting that higher concentration of 5-aza could promote the differentiation. We also observed that in the combination of 0.1 µM Vc and 5 µM 5-aza percentage of beating cell masses (33%) was significantly higher than that in the combination of 0.1 µM Vc and 10 µM 5-aza, indicating that Vc might play a key role in the induction (Fig. 5C). The results of transmission electron microscope revealed that beating cell masses had the basic structures of myocardium, such as myofilament and glycogen granule (Fig. 5D), although the Z line was not yet observed.

## Discussion

For prenatal diagnosis, most amniocenteses are performed in the early week gestation. Within the amniotic fluid are fetal cells which can be grown in culture for chromosome analysis, biochemical analysis, and molecular biologic analysis [20]. Gosden et al. reported that the cells existing in AF were originated from development embryos, which contained a mixture of multiple cell types [7], [21]. In our study, we found that both epithelial-like and fibroblast-like cells appeared in the primary porcine AF culture. After several passages, most cells exhibited fibroblast-like appearance. This might imply that fibroblast-like cells were the dominant stem cells in AF. Therefore, some researchers named these cells as AF-mesenchymal stem cells [22]. The specimen from 2–5 ml of amniotic fluid was enough to generate the primary culture. After the subculture for 3–4 passages in vitro, the cell numbers could reach 0.8×10^6^ per 3.5 cm plate. Over 7 passages, there 2.1×10^7^ cells were obtained, which were either frozen or used to cellular experiments. The optimal viability and proliferation of pAF-MSCs was from 8^th^ passage till 30^th^ passage, though one pAF-MSC line was cultured for more than 60 passages in vitro.

Recently, many reports suggested that hAFS cells were a group of multipotent stem cells with a great promise in cell therapy and tissue engineering [7], [23]. But so far, very few studies were reported on porcine AFS. The pAF-MSCs were easy to culture and expressed specific surface antigens including CD117, CD44, CD90, CD166 and HLA-abc, but not CD45, CD34 and CD54. The most gene expression profiles of pAF-MSCs were similar to hAFS cells [4]. However, the expressions of Tra-1-60 and Tra-1-81 were not found in hAFSCs [24], while pAF-MSCs expressed both Tra-1-60 and Tra-1-81 markers. These differences might be caused by the species difference since we did find the sub-group of cell population with CD117−/CD44+ and CD117+/CD44+ in human amniotic fluid stem cells (unpublished data). The results to monitor the expression of CD90 and CD117, the mesenchymal stromal cell markers, in the different passages showed that these markers were constantly expressed in pAF-MSCs, although the expression levels were slightly decreased in later passages (Fig. 2B). Thus, these two genes plus CD44 could be used as the pAF-MSCs markers. Besides, the hematopoietic cell marker CD45 and CD34 were not detected in pAF-MSCs, suggesting that pAF-MSCs did not come from the hematopoietic cell derivative. Additionally, pAF-MSCs expressed the transcription factors Oct4, Nanog and cell surface marker SSEA4, which were the important regulators for maintaining the pluripotent state in mES and hES cells [25], [26], [27], [28]. The above features indicated that the pAF-MSCs were in an intermediate state between pluripotent ES cells and lineage-restricted adult stem cells.

It was reported that the stem cells isolated from porcine AF had the ability of neurogenic differentiation [29]. However, the ability to differentiate into cardiogenic phenotypes in an autotransplanted model of cardiac ischemia was not achieved [30]. In this study, the pAF-MSCs could express markers of three germ layers and differentiate into myocardial cell mass. Under the treatment of Vc and 5-aza, cell mass from pAF-MSCs could beat rhythmically, symbolizing the cardiac muscle. More experiments are needed to determine if these beating cells can be used for the therapy of cardiac ischemia in pig model. The embryoid body formation mimics the earliest stages of embryonic development. Many protocols of embryonic stem cell differentiation have been reported to depend on EBs to initiate the spontaneous differentiation that occurs within 3-dimensional aggregates [19]. In the beginning experiments, we have tried both EB and monolayer methods to induce pAF-MSCs into cardiomyocytes. The result indicated that the induction efficiency by monolayer method was low, and the induced products were difficult to characterize due to the less beating cell population.

One merit of hAFSCs was their non-tumorigenesis, which was different from hES cells [7]. In our experiment, we also confirmed that the injection of pAF-MSCs into the immunodeficient mice didn't generate tumors. This result suggested that pAF-MSCs cells were the valuable seeded cells that could be used for the transgenic animals and as the recipient cells for gene knockout.

In conclusion, we successfully isolated and characterized pAF-MSCs from porcine amniotic fluid. They possessed self-renewal and multi-lineage differentiation potency, which could differentiate into neuroncytes, adipocytes and myocardial cell mass. The pAF-MSCs would provide an ideal cell resource for regenerative medicine and tissue engineering. Moreover, the molecular mechanisms involved in the self-renewal and multipotency of pAF-MSCs remains unclear and need to be further investigated.

## Materials and Methods

### Isolation and growth of pAF-MSCs

The pig amniotic fluid (2–5 ml) was collected from the amniotic cavity of pregnant crossbred gilt in the early stage of gestation (at E35) and centrifuged at 400 g for 5 min. After three times washing with PBS, the cells were gently suspended and plated in 6-well culture plate (Costar) with pAF-MSC medium (AMM), including α-MEM medium (Invitrogen, all the reagents used without emphasized were from Invitrogen.), 15% FBS, 1% glutamine and 1% penicillin/streptomycin, at 38°C in 5% CO_2_ incubator. After 3 days growth, both fibroblast-like cells and epithelioid cells appeared and formed cell clusters. The epithelioid cells were removed by Tissue Cell Scraper, and fibroblast-like cell clusters were sub-cultured in new 12-well plates (Costar). This process was repeated twice. Finally, the uniform fibroblast-like cells were cultured in 3.5 cm culture plate. When reached 80% confluence (0.8×10^6^ cells/plate), cells were treated with 0.05% Trypsin-EDTA and passaged every two days with a 1∶3 split. To determine the growth rate of cells, the pAF-MSCs at 6^th^ passages were plated at 4×10^3^ in each well of the 24-well plate and cultured for 8 days. Cells in every three wells were collected from day 1 to day 8 and counted using a hemocytometer. The average cell numbers for each three wells were calculated and the growth curve was plotted with the average number of cells at different times.

### Flow cytometry analysis

The DNA content and surface antigens of pAF-MSCs were examined by flow cytometry analyses. To detect DNA content, cells were harvested by the treatment of Trypsin and fixed in 70% ethanol. After incubation in an ice-bath for 30 min, the cells were washed three times with washing buffer (PBS with 1% BSA and 0.05% NaN_3_), and then resuspended in 20 ml of washing buffer and 10 ml of Propidium Iodide (PI) solution (stock solution 10 mg/ml). Approximately 1×10^6^ cells/ml were used for the flow cytometry cell sorting (Beckman Coulter). To determine the surface antigens of pAF-MSC, cells were trypsinized and stained with fluorescein isothiocyanate (FITC)-conjugated or phycoerythrin-conjugated antibodies against CD117 (559879#, BD, USA), CD34 (555823#, BD, USA), CD166 (Santa, USA), CD54 (Santa, USA), CD44 (555478#, BD, USA), and CD45 (555482#, BD, USA). The cells were then analyzed by flow cytometry (Becton Coulter).

### Total RNA extraction and RT-PCR

Total RNA of pAF-MSCs was extracted using Trizol reagent according to the manufacturer's instruction, and high quality RNA (OD260/280 = 2.0) was used for the reverse transcription reaction. RT-PCR was performed according to a coupled one-step procedure using Access RT-PCR System (Promega, Madison WI, USA). Briefly, 2 µg of total RNA was reverse transcribed at 37°C for 1 h, denatured at 94°C for 2 min, and amplified for 30 cycles of denaturation at 94°C for 30 sec, primer annealing at 55°C for 30 sec, and extension at 72°C for 45 sec, followed by a final extension step at 72°C for 10 min. The amplified products were analyzed by electrophoresis on 1% agarose gel. Images were taken with a High Performance CCD camera. The primers used in this experiment were listed in Table 1.

**Table 1:** Primer sequences for RT-PCR.

Primer	Sequences, 5′ to 3′	Size (bp)	Accession number
β-actin	S: TACGACTGGCATTGTGC	360	EU655628
	A: TACGACTGGCATTGTGC		
CD117	S: ACCGCACTGCCACTGAT	427	NM_001044525
	A: TAAGCCCTGCACTCCAC		
CD90	S: GACCCGTGAGACAAAGCAGC	171	DQ400919
	A: TGGCCAGAGTGGTGGAGTTC		
CD45	S: CCTCCAGATCCTACAAA	443	AY444867
	A: CCATCACTCCGATAACA		
CD56	S: GAGGGGGAAGATGCCGTGATGTG	269	NC_006587.2
	A: ATATTCTGCCTGGCCCGGATGGTAG		
AFP	S: GGAGAAATGTTCGCAGTC	306	NM_214317.1
	A: TGCCCGATGATAAGGT		
Bra	S: AAGAACGGCAGGAGGATGG	372	XM_001928144.1
	A: CTCTGGGAAGCAGTGGC		
FGF5	S: TCACGGGGAGAAGCGTCT	407	NM_004464.3
	A: ACTTGGCACTTGCATGGA		
HLA-abc	S: GTATTTCTTCACATCCGTGTCCCG	394	M27971.1
	A: GTCCGCCGCGGTCCAAGAGCGCAG		
PPARγ	S: GATGCCACAGGCCGAGAAGG	235	NC_000003.10
	A: GCCCTGAAAGATGCGGATGG		
C/EBPα	S: CCCCGCGAGGAGGATGAAGC	126	NC_000019.8
	A: GCACCCGGTACTCGTTGCTGTTCT		
TbX5	S: ATTGCTGAAACCGAGAATGG	250	AM937230.1
	A: GCGCTCCTTGAGGTTGAAAAG		
Gata4	S: CTGTGCCAACTGCCAGACCA	437	NM_214293.1
	A: GGCTGACCGAAGATGCGTAG		
α-MHC	S: AGAAGATAGTGGAACGCAG	147	NC_000014.8
	A: GCATCATTGAGGTTGTCTTG		
TNNi3	S: GAGTGAGGATCTCTGCAGAT	130	NM_000363
	A: GATGTTCTTGCGCCAGTCTC		
MYL	S: CTCCAACGTGTTCTCCATG	272	NM_000432
	A: CCTTGAATGCGTTGAGAATG		
NKX2.5	S: CATGCTGGCCGCCTTCAAG	438	NM_004387
	A: CCAGATCTTGACCTGCGTG		

### Immunocytochemistry

The cells were grown at 37°C in a humidified CO_2_ incubator until they were 60–70% confluent, and were then fixed in 4% paraformaldehyde solution and blocked with 5% bovine serum albumin (BSA) for 1 h. The blocked cells were incubated with primary antibody at a dilution of 1∶100 at 4°C overnight. After three times washing, the secondary antibody conjugated with either FITC or TRITC was added, respectively, at a dilution of 1∶100 in PBS for 1 h at 37°C. Nuclei were stained with Hoechst 33342 (10 ug/ml) for 1 min and viewed under a laser scanning Confocal microscope (Leica, Heidelberg, Germany). In some experiments, biotinylated secondary antibodies and an avidin-biotinylated enzyme complex system (Vectastain Elite ABC Kits) with a DAB substrate kit (Vector Laboratories) were used to visualize positive cells. For negative control, parallel experiments were performed with cells using pre-immune serum of the corresponding animal. The primary antibodies used in this experiment were as below: anti-Oct4 (ab13840, Abcam, UK), anti-Nanog (ab21603, Abcam, UK), anti-SSEA1 (90230, Millipore, USA), anti-SSEA4 (90231, Millipore, USA), anti-Tra-1-60 (90232, Millipore, USA), anti-Tra-1-81 (90233, Millipore, USA), anti-NF-200 (ab82259, Abcam, UK), anti-Nestin (MAB5326, Chemicon), anti-TERT (sc-7212, Santa Cruz, UAS), anti-α-actin (A2172, Sigma, USA), anti-β-tublin (bs-0210R, Beijing Biosynthesis Biotechnology Co, LTD, China ), anti-Nkx2.5 (ab97355, Abcam, UK).

### Embryoid bodies and Teratoma formation

The pAF-MSCs at the 12^th^ passage were harvested with 0.05% Trypsin and reseeded in the ultra-low binding dish with a concentration of 1×10^6^ cells/ml. During the 24 h incubation in the suspension culture, embryoid bodies (EBs) were formed, and continuously grew for 2–3 days allowing to form bigger and even EBs. To detect alkaline phosphatase activity, the EBs from pAF-MSCs were fixed with 4% paraformaldehyde for 10 min at room temperature, washing twice with ice-cold PBS, EBs were then incubated with Fast Red TR/Naphthol AS-MX Phosphate (Fast Red TR 1.0 mg/ml, Naphthol AS-MX 0.4 mg/ml, in 0.1 M Tris Buffer) for 20–30 min at room temperature. A red color stain indicated the positive reaction for alkaline phosphatase. The images were photographed by Leica 1300 digital CCD camera (Roper Industries, Duluth, GA).

The teratoma formation assays with pAF-MSCs were conducted in Animal Center of the Fourth Military Medical University, which was the licensed animal research facility and provides the commercial services. The pAF-MSCs (5×10^6^ cells/injection) were subcutaneously injected into the right axilla of three immunodeficient mice (BALB/c-Nu). One mouse injected with mES J1 cells (2×10^6^ cells/injection) was a positive control. The mice breeding and cell injection experiments were carried out by Fourth Military Medical University in Xi'an, Shaanxi, China. Post-injection for 45 days, the teratoma tissue was collected, and used for the histochemistry assay and RNA extraction.

### Differentiation of pAF-MSCs into three germ layer lineages

To induce pAF-MSCs into three germ layer lineages, cells at the 12^th^ passage were harvested with 0.05% Trypsin and reseeded in ultra-low binding dish with a concentration of 1×10^6^ cells/ml to form EBs for 3 days. The EBs were then transferred to gelatin-coated dishes to attach, and grew in the induction media to induce cell differentiation. After the induction, the tissue specific gene markers were then examined to define the progression of the differentiation by the immunocytochemy (ICC) and RT-PCR analysis. To perform adipogenic differentiation, EBs were cultured in adipogenic differentiation medium (α-MEM supplemented with 1 µM dexamethasone, 0.5 mM 3-isobutyl-1-methylxanthine (IBMX), 1.7 µM insulin, and 0.2 mM indomethacin) for two weeks, changing medium every 2–3 days. Oil Red O staining was used to detect adipocytes. To perform neurogenic differentiation, cells were cultured in neurogenic differentiation medium (a-MEM supplemented with 20% FBS, 1 mmol/l b-mercaptoethanol, 5 ng/ml bFGF) for 24 h, and then treated with serum depletion medium for 5 h. The neuron specific markers of Nestin, NF-200, FGF5 and CD56 were examined. After the cytochemistry determination, the images were photographed by Leica 1300 digital CCD camera (Roper Industries, Duluth, GA).

To perform cardiac differentiation, we made five different media, which included 1# AMM+0.1 mM Vc; 2# AMM+5 µM 5-aza; 3# AMM+10 µM 5-aza; 4# AMM+0.1 mM Vc+5 µM 5-aza; and 5# AMM+0.1 mM Vc+10 µM 5-aza. The control medium was AMM. To differentiate pAF-MSCs into beating cardiomyocytes, cells were cultured in suspension in AMM for 5 days to form EBs, and then EBs were pelleted and resuspended in media 1#–5#, respectively, and continually grew in suspension for 2 days. After total 7 day in suspension, cells were transferred into 48-well tissue culture plate allowing EBs to attach the plate surface, and grew in fresh AMM for 10 days, changing medium in every 2–3 days, to induce the beating cardiomyocytes. The percentage of beating cell masses was counted. The experiments were repeated separately for 3 times. The differentiation efficiency was compared via statistical analysis where p<0.05 was considered statistical significant. To perform the immunocytochemistry assay, the beating cell masses were treated with 0.05% Trypsin, reseeded on a new plate for 12 h, and then ran the assay described above. To conduct the histochemistry analysis, the rhythmically beating cell masses were picked out and fixed by 3% glutaraldehyde. The transmission electron microscopy analysis was carried out by the Central Laboratory of Fourth Military Medical University (Xi'an, Shaanxi, China).

## Supporting Information

Figure S1 **The beating cardiomyocyte-like cell clusters differentiated from pAF-MSCs.** (A) The fast beating cell mass. The rate of beating frequency was in the range of 90–110 beats/min. (B) The slow beating cell mass. The rate of beating frequency was in the range of 25–50 beats/min. (C) The monolayer beating cells.
(TIF)

Movie S1 The fast beating cell mass.
(MP4)

Movie S2 The slow beating cell mass.
(MP4)

Movie S3 The monolayer beating cells.
(MP4)
