# Supplementary figures and images for: Isolation and Characterization of Porcine Amniotic Fluid-Derived Multipotent Stem Cells
Source: PLoS One. 2011 May 19;6(5):e19964. doi: 10.1371/journal.pone.0019964 (PMC3098286; doi:10.1371/journal.pone.0019964)

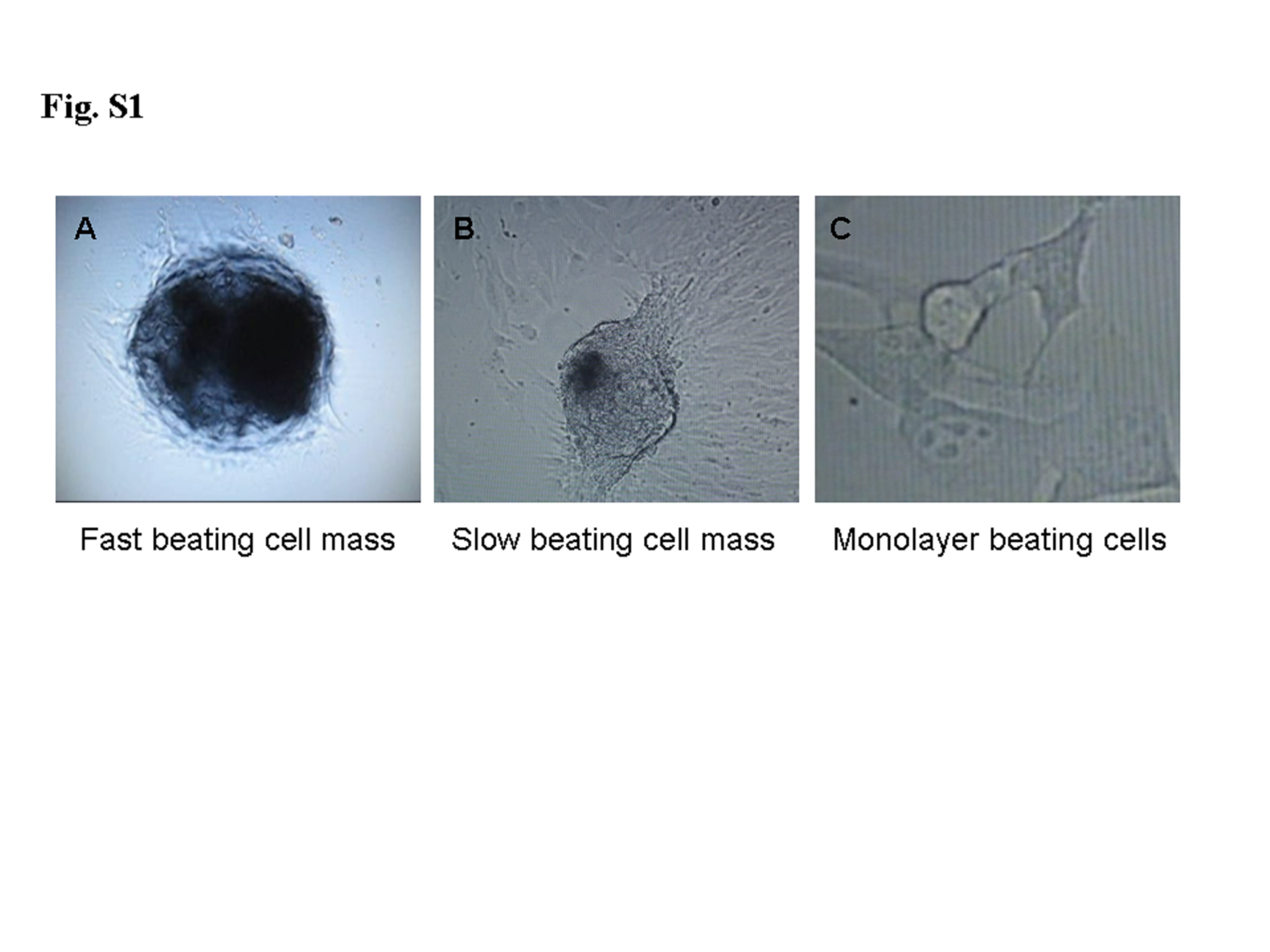

Supplement: Figure S1 — The beating cardiomyocyte-like cell clusters differentiated from pAF-MSCs. (A) The fast beating cell mass. The rate of beating frequency was in the range of 90–110 beats/min. (B) The slow beating cell mass. The rate of beating frequency was in the range of 25–50 beats/min. (C) The monolayer beating cells. (TIF) [file pone.0019964.s001.tif]
